# Comparative chloroplast genomics of six *Bupleurum* (Apiaceae) accessions: candidate barcodes, phylogeny based on available plastomes, and candidate RNA-editing sites

**DOI:** 10.3389/fpls.2026.1916637

**Published:** 2026-08-20

**Authors:** Yujie He, Mingxuan Wu, Aohan Wang, Chengwen Gao

**Affiliations:** Medical Research Center, The Affiliated Hospital of Qingdao University, Qingdao University, Qingdao, Shandong, China

**Keywords:** Apiaceae, *Bupleurum*, candidate molecular marker, phylogenomics, plastome, RNA editing

## Abstract

**Introduction:**

*Bupleurum L*. (Apiaceae), a taxonomically intricate genus of about 190 species and a source of Radix Bupleuri (Chai Hu), is difficult to discriminate because of convergent morphology, infraspecific variation, and limited genomic sampling. This study aimed to characterize plastome variation, identify and validate candidate molecular markers, reconstruct plastid phylogenetic relationships, and assess candidate plastid RNA-editing sites in *Bupleurum*.

**Methods:**

We assembled six plastomes from subgenus *Bupleurum*, screened 51 *Bupleurum* plastomes for diagnostic loci, reconstructed whole-plastome and partitioned protein-coding-sequence phylogenies, and predicted plastid C-to-U RNA-editing candidates across the six newly assembled plastomes using a PREP-Cp-compatible workflow. Candidate barcode performance was evaluated against the reference plastome phylogenies, and codon-based models were used to test for positive selection.

**Results:**

The plastomes were 154,496–155,778 bp with the canonical quadripartite structure and GC contents of 37.67–37.73%. Gene content was stable (131–132 genes; 86–87 protein-coding genes); *B. falcatum* subsp. *cernuum* lacked *ycf15* but contained an additional inverted-repeat-associated *ycf1* annotation. A/U-ending synonymous codons were favoured. Finite pairwise Ka/Ks estimates were below 1 for most genes, and site-specific codon models detected no positive selection. Each plastome contained 55–61 pure microsatellites, dominated by A/T mononucleotide motifs. MarkerSeek ranked 265 features and identified *atpF–atpH*, *petA–psbJ*, *rpl32–trnL-UAG*, and *ycf1* as leading candidate barcodes. *ycf1* recovered 38 of 41 nodes strongly supported by both reference trees, whereas a partitioned four-locus analysis recovered 40 of 41 and distinguished all 51 accession sequences. However, only one of seven multi-accession operational binomial groups was monophyletic, and only one showed a positive local barcode gap. The whole-plastome phylogeny recovered *Bupleurum* as monophyletic relative to *Chamaesium*. The two sampled *Penninervia* accessions occupied early-diverging positions without forming an exclusive clade. *B. falcatum* subsp. *cernuum* was sister to *B. ranunculoides*, with *B. ranunculoides* subsp. telonense sister to that pair. A partitioned 74-CDS analysis recovered the same key relationships and 45 of 50 internal bipartitions. Across the six newly assembled plastomes, 57–63 nonsynonymous C-to-U candidates were predicted per accession (367 total) in 21–22 genes; 269 affected the second codon position and 98 the first.

**Discussion:**

*Bupleurum* plastomes are structurally conservative but retain localised divergence useful for marker development. Concordant whole-plastome and CDS genealogies support genus monophyly, whereas sparse *Penninervia* sampling and maternal plastid inheritance preclude rejecting traditional subgeneric classification. The predicted RNA-editing sites represent candidates for future experimental validation rather than an established *Bupleurum* editome. These genomic resources support authentication, conservation, and evolutionary research in *Bupleurum*.

## Background

*Bupleurum* L. is one of the larger and more widely distributed genera of Apiaceae, comprising roughly 190 species of herbs and a few woody perennials that radiate across the temperate Northern Hemisphere, with a centre of diversity in the mountains of southwestern China and a secondary concentration around the Mediterranean basin (Neves and Watson, 2004; Song et al., 2024). The genus is also of considerable applied interest. Dried roots of several species, traded as Radix Bupleuri, have been used for centuries to treat fever and inflammatory and hepatic disorders, and the saikosaponins responsible for much of this activity continue to attract pharmacological attention (Teng et al., 2023; Sun et al., 2024; Gu et al., 2025). Because closely related taxa are frequently substituted in the herbal supply chain, accurate identification of *Bupleurum* species and their products carries direct consequences for efficacy and safety (Wang et al., 2023).

Delimiting species within *Bupleurum* is, however, notoriously difficult. The simple, entire, parallel-veined leaves that characterise most of the genus offer few stable diagnostic characters, vegetative plasticity blurs the boundaries between morphologically similar taxa, and several widespread species encompass a confusing array of subspecies and varieties (Neves and Watson, 2004). Current infrageneric classification recognises two subgenera, the largely Mediterranean and woody subgenus *Penninervia*, distinguished by pinnately reticulate venation, and the species-rich subgenus *Bupleurum* with parallel leaf venation (Neves and Watson, 2004). Molecular work based on nuclear ribosomal and a few plastid loci has confirmed the monophyly of the genus and supported its segregation as the tribe Bupleureae, but relationships among the rapidly radiating East Asian lineages have remained only partly resolved (Chao et al., 2023; Song et al., 2024).

Plastid genomes are well suited to addressing problems of this kind. They are typically small, structurally conserved, predominantly maternally transmitted, and present in high copy number, so that complete sequences can be recovered economically from low-coverage genome skimming and then mined simultaneously for fine-scale markers and deep phylogenetic signal (Wicke et al., 2011; Ruhlman and Jansen, 2021). In Apiaceae, comparative plastome studies have repeatedly proven informative, not least because the family displays unusually dynamic expansion and contraction of the inverted repeat that can itself carry taxonomic information (Yuan et al., 2021; Joh et al., 2025). For *Bupleurum*, plastomes have so far been assembled mainly for southwestern Chinese and a few Mediterranean species, and these efforts have begun to point to a small number of rapidly evolving intergenic regions as potential identification tools (Huang et al., 2021a; Huang et al., 2021b; Huang et al., 2022; Chao et al., 2023). A coordinated analysis that couples broad taxon sampling with an explicit, repeatable procedure for ranking candidate markers has nonetheless been lacking.

A further layer of plastid biology that has not, to our knowledge, been examined in *Bupleurum* is RNA editing. In seed-plant plastids, editing is dominated by the site-specific deamination of cytidine to uridine, a process that frequently restores evolutionarily conserved codons and can modulate the function of the edited proteins (Small et al., 2020; Knoop, 2023). Editing inventories vary among lineages and are known experimentally for only a small fraction of sequenced plastomes, so a conservatively filtered candidate inventory can guide subsequent experimental validation (Ichinose and Sugita, 2017).

Here we combine six newly assembled *Bupleurum* plastomes from subgenus *Bupleurum* with a published data set of available *Bupleurum* plastomes to pursue four aims: (i) to describe genome organisation, gene content, codon usage and pairwise substitution-rate patterns in the six new plastomes; (ii) to screen a broader 51-accession data set systematically for candidate molecular markers using a transparent, score-based pipeline and to evaluate four leading candidates through single-locus and partitioned multilocus phylogenetic analyses; (iii) to reconstruct plastid relationships across both recognised subgenera from complete plastomes and test the topology with a partitioned protein-coding-sequence data set; and (iv) to generate a comparative in silico inventory of candidate plastid C-to-U editing sites across the six newly assembled plastomes. The resulting resources are intended to support species authentication, germplasm management and continued evolutionary study of *Bupleurum*.

## Methods

### Taxon sampling, sequencing data and plastome assembly

Six *Bupleurum* accessions from subgenus *Bupleurum* were selected across a range of geographic origins: *B. arcticum*, *B. diversifolium*, *B. falcatum* subsp. *cernuum*, *B. longifolium*, *B. ranunculoides* and *B. ranunculoides* subsp. *telonense*. The *B. arcticum* sample consisted of herbarium leaf material, whereas the other five samples consisted of silica-gel-dried fresh leaf material; accession-specific collection localities, dates, collectors, sample or voucher identifiers, and voucher deposition where reported are provided in **Supplementary Table 1**. Illumina sequencing reads were obtained from public whole-genome sequencing datasets (**Supplementary Table 1**). Raw read-pair counts, total sequenced bases and source-reported plastome coverage are also given in **Supplementary Table 1**. Reads were adapter- and quality-trimmed with Trimmomatic v0.38 using ILLUMINACLIP: TruSeq3-PE, LEADING:3, TRAILING:3, SLIDINGWINDOW:4:15 and MINLEN:50 (Bolger et al., 2014). Plastid-enriched read pairs were selected by Bowtie2 mapping to a closely related *Bupleurum* plastome (Langmead and Salzberg, 2012) and assembled with SPAdes v3.6.1 in --careful mode with eight threads and requested k-mer sizes of 55, 75, 95, 105 and 125; k-mer sizes exceeding the read length were skipped automatically (Bankevich et al., 2012). Chloroplast scaffolds were ordered and oriented against the closest available reference, and the resulting circularized sequences and inverted-repeat junctions were checked by self-alignment. Plastomes were annotated with CPGAVAS2 (Shi et al., 2019), annotations and region boundaries were inspected against homologous plastomes, and circular maps were drawn with OGDRAW (Greiner et al., 2019). GenBank accession numbers PZ518415–PZ518420 have been assigned to the six plastomes.

### Codon usage, selective pressure and repeat content

**Figure 1:**
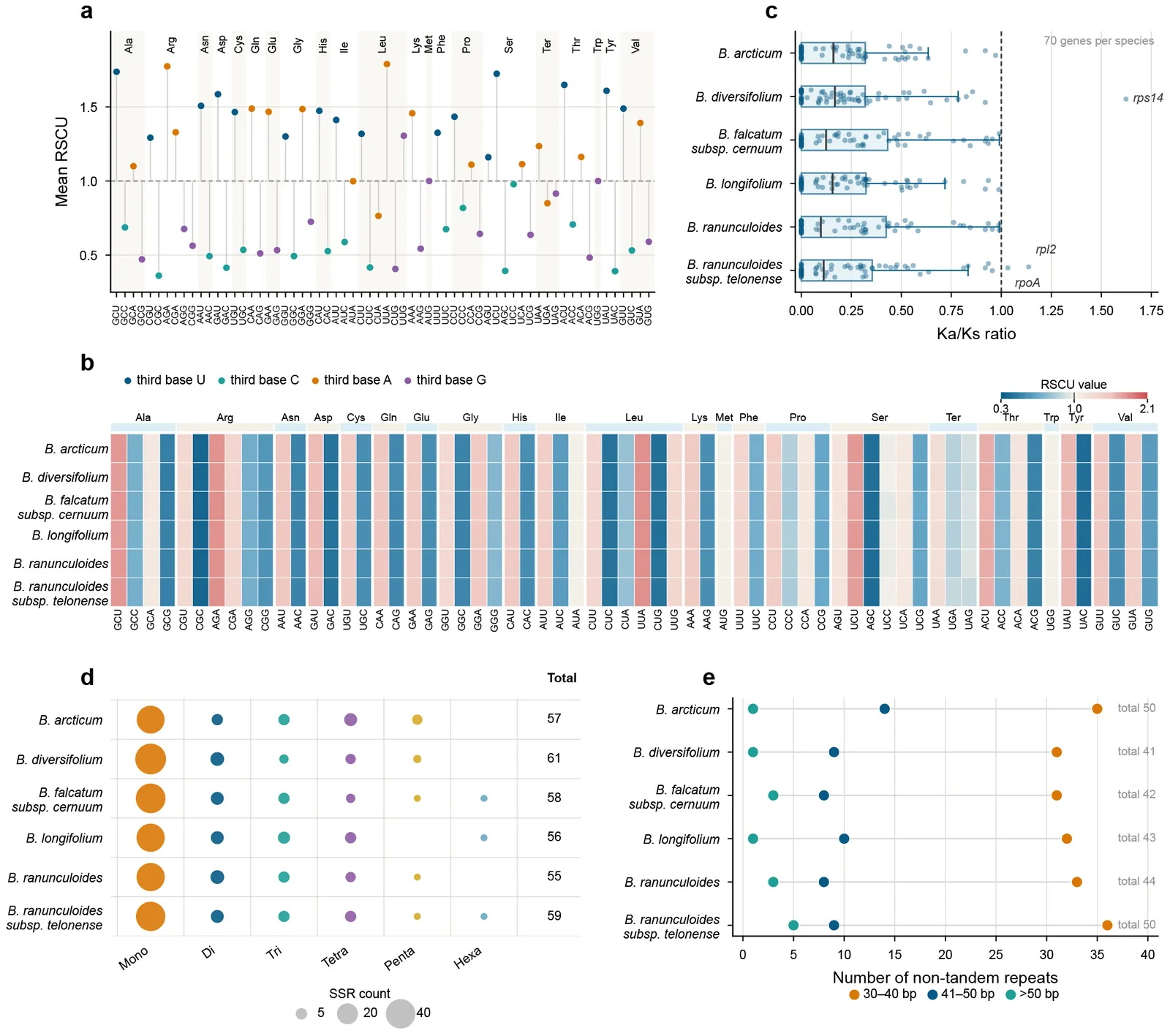
Codon usage, pairwise Ka/Ks estimates and repeat content of the six *Bupleurum* plastomes. **(A)** Mean relative synonymous codon usage (RSCU) by amino acid. **(B)** Hierarchically clustered RSCU heatmap. **(C)** Gene-wise Ka/Ks estimates relative to *B. chinense*; undefined cells were replaced by within-gene means for visualization only, the dashed line marks Ka/Ks = 1, and directly estimated outliers are labelled. **(D)** Pure SSR counts by motif class; compound SSRs are excluded. **(E)** Forward and palindromic repeat pairs by size class; the >50-bp class includes the full-length inverted-repeat match.

Relative synonymous codon usage (RSCU) was computed for the concatenated protein-coding genes of each plastome following the standard formulation (Sharp and Li, 1986); codons with RSCU above 1 were treated as favoured. Every spliced protein-coding gene was first checked against the canonical group II intron boundaries (5′ GUGYG, 3′ AY). In all six new plastomes the *atpF* exon 1/exon 2 junction had been placed 18 bp downstream of the canonical 3′ splice site, so that six codons of intron were retained in the coding sequence; *atpF* was therefore re-annotated (exon 1, 141 bp; exon 2, 462 bp; coding sequence, 603 bp) and all *atpF* comparisons use the re-annotated sequences. No other spliced gene required correction. Orthologous coding sequences were aligned in frame, and non-synonymous (Ka) and synonymous (Ks) substitution rates and their ratio were estimated relative to *B. chinense* (NC_046774.1) with KaKs_Calculator 3.0 using the MLWL method (Zhang, 2022). Undefined ratios were retained as missing values in **Supplementary Table 4**. For visualization in **Figures 1C** only, missing cells were replaced by the mean of the finite estimates for that gene and were not used to identify ratios above 1. Because pairwise Ka/Ks ratios are unstable when synonymous substitutions are few, *atpF* and the three genes whose pairwise ratios exceeded 1 (*rps14*, *rpoA* and *rpl2*) were also examined with site-specific codon models. In-frame codon alignments of the same 53 taxa used for the phylogenomic analyses were prepared with MAFFT v7.525, terminal stop codons were removed and internal stop codons were recoded as missing data. Using the partitioned 74-CDS topology as the input tree, site-wise synonymous (α) and non-synonymous (β) rates were estimated with a fixed-effects likelihood model (FEL) and episodic diversifying selection was tested with a mixed-effects model of evolution (MEME) in HyPhy 2.5.65 (Kosakovsky Pond and Frost, 2005; Murrell et al., 2012; Kosakovsky Pond et al., 2020); sites are reported at p ≤ 0.05. Simple sequence repeats (SSRs) were detected with MISA (Beier et al., 2017) using minimum repeat counts of 10, 5, 4, 3, 3 and 3 for mono- through hexanucleotide motifs, respectively. Forward and palindromic non-tandem repeats were identified with REPuter (Kurtz et al., 2001) using a minimum repeat length of 30 bp and a Hamming distance of 3.

### Comparative divergence and candidate-marker screening

**Figure 2:**
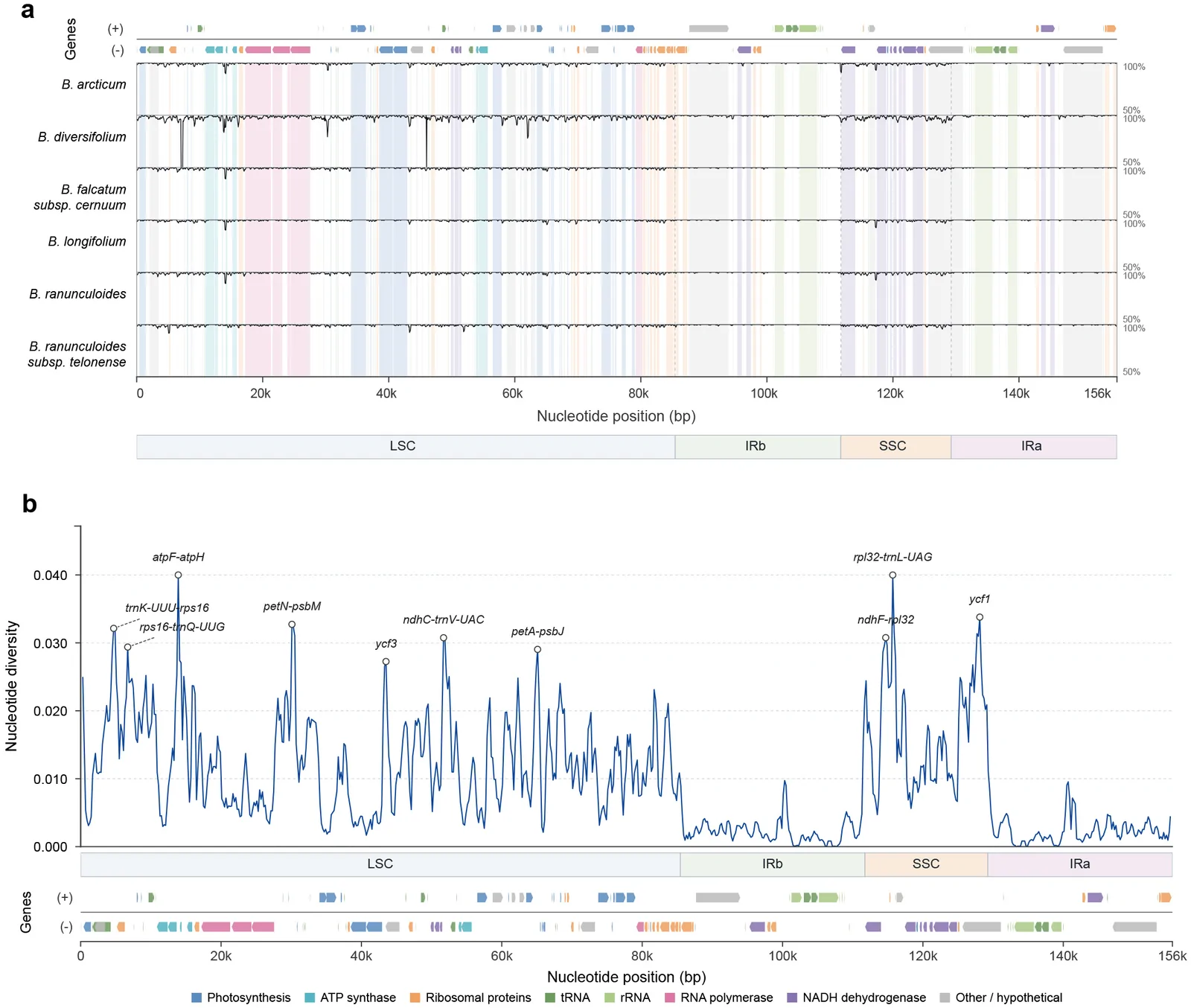
Comparative divergence and candidate-marker screening. **(A)** Sequence identity among the six newly assembled *Bupleurum* plastomes. **(B)** Sliding-window nucleotide diversity (π; 600-bp window, 200-bp step) across 51 *Bupleurum* plastomes, with high-scoring MarkerSeek regions labelled. Plastome regions (LSC, IRb, SSC and IRa) and gene tracks are shown.

Sequence identity among the six new plastomes was displayed in **Figure 2A**. A broader 51-accession *Bupleurum* data set comprising 45 public plastomes and the six new plastomes was compared with MarkerSeek using *B. chinense* as the reference (**Supplementary Figure 1**). Candidate molecular markers were screened across the same 51 accessions with MarkerSeek, a tool we developed for plastid candidate-marker discovery (https://github.com/gaochengwen/MarkerSeek) (Gao, 2025). MarkerSeek aligns annotated plastomes with MAFFT (Katoh and Standley, 2013), projects genes, tRNAs, rRNAs and intergenic spacers onto the reference coordinate system, and estimates nucleotide diversity (π) in 600-bp windows advanced in 200-bp steps (Nei and Li, 1979). Each feature is scored from 0 to 100 using diversity, variable- and indel-site density, conserved flanks, alignment reliability and species-level resolution. Because most nominal taxa were represented by a single accession, replicate-dependent diagnostics were unavailable for the MarkerSeek ranking and their weights were redistributed across the remaining terms.

### In silico evaluation of candidate barcodes

The three top-ranked intergenic spacers (*atpF*–*atpH*, *petA*–*psbJ* and *rpl32*–*trnL-UAG*) and the leading coding candidate (*ycf1*) were extracted from the same 51 *Bupleurum* plastomes and two *Chamaesium* outgroups. For *ycf1*, the longest annotated copy was retained to exclude the short inverted-repeat-boundary copy. Each locus was aligned independently with MAFFT v7.505 using --auto (Katoh and Standley, 2013), and single-locus maximum-likelihood trees were inferred with IQ-TREE v2.0.7 under the BIC-selected ModelFinder model and 1,000 ultrafast-bootstrap replicates (Kalyaanamoorthy et al., 2017; Hoang et al., 2018; Minh et al., 2020; Wong et al., 2026). The four alignments were also concatenated and analysed under an edge-linked proportional partition model with models selected independently by partition (Chernomor et al., 2016; Wong et al., 2026). We recorded alignment length, variable and parsimony-informative sites, gaps or ambiguous characters, bootstrap support, and recovery of unrooted bipartitions in the whole-plastome and 74-CDS reference trees (Robinson and Foulds, 1981). For the 51 ingroup accessions, candidate discriminatory performance was summarised as exact sequence uniqueness, identical sequence pairs and nearest-neighbour uncorrected p-distances with pairwise deletion; strict monophyly and local barcode gaps (Meyer and Paulay, 2005) were evaluated only for the seven operational binomial groups represented by multiple accessions. These analyses assess in silico phylogenetic resolution and candidate discriminatory performance, not validated species identification (**Supplementary Table 10–S12**).

### Phylogenomic reconstruction

The six new plastomes were combined with 45 published *Bupleurum* plastomes spanning both recognised subgenera, and two *Chamaesium* species (*C. paradoxum*, MK780227; *C. spatuliferum*, MN119371) were used as outgroups, giving 53 taxa (**Supplementary Table 9**). *Chamaesium* is a closely related non-*Bupleurum* apioid lineage used to root recent genus-level analyses (Song et al., 2024); two representatives were included here to reduce dependence on a single outgroup accession. Complete plastome sequences were aligned with MAFFT v7.490 using the --auto strategy (Katoh and Standley, 2013). Maximum-likelihood inference was performed in IQ-TREE 3.0.1 (Wong et al., 2026); ModelFinder selected TVM+F+R5 under the Bayesian information criterion (Kalyaanamoorthy et al., 2017), and branch support was assessed with 1,000 ultrafast-bootstrap replicates (Hoang et al., 2018). As a sensitivity analysis that avoids dependence on plastome linearization and non-coding sequence alignment, protein-coding sequences were extracted from GenBank annotations; one copy of each inverted-repeat duplicate was retained, the trans-spliced *rps12* was excluded, and 74 CDS present in at least 52 taxa were aligned separately with MAFFT. The resulting 66,250-bp supermatrix was analysed by partitioned maximum likelihood in IQ-TREE 3.0.1 (Chernomor et al., 2016; Wong et al., 2026), with ModelFinder selecting a model for each gene partition and 1,000 ultrafast-bootstrap replicates. Both trees were rooted with the two *Chamaesium* accessions, and newly assembled taxa were highlighted.

### Prediction of candidate plastid RNA-editing sites

Candidate plastid C-to-U RNA-editing sites were predicted for the six newly assembled plastomes using an auditable local implementation of the published PREP-Cp conservation-based procedure (Mower, 2005; Mower, 2009). The analysis was restricted to the 35 protein-coding genes supported by PREP-Cp and used the recommended score cutoff of 0.80 (Mower, 2009). Spliced coding sequences were translated and aligned with MAFFT v7.505 (Katoh and Standley, 2013) against mature homologous proteins reconstructed from the seven PREP-Cp training taxa (Mower, 2009). Curated editing annotations for *Arabidopsis thaliana*, *Nicotiana tabacum*, *Atropa belladonna*, *Zea mays*, *Oryza sativa* and *Phalaenopsis aphrodite* were obtained from PREPACT 3.0 (Lenz et al., 2018), and experimentally verified sites were applied to *Pinus thunbergii* (Wakasugi et al., 1996). Reference proteins shorter than 50% of the target protein were excluded gene by gene.

At each nonsynonymous genomic C, the translated C-to-U alternative was scored as the proportion of aligned, non-gap reference residues matching the edited amino acid; a site was retained when the edited residue was more frequent than the unedited residue and its score was at least 0.80 (Mower, 2005; Mower, 2009). Because the original PREP-Cp server and its manually adjusted alignments are no longer available, this auditable reconstruction used MAFFT v7.505 and is described as PREP-Cp-compatible rather than as exact server output. The archived *Vitis accD* example was used as a regression check, and per-site scores, reference-residue counts and alignments were retained for audit (**Supplementary Table 13**).

## Results

### Plastome organisation and gene content

**Figure 3:**
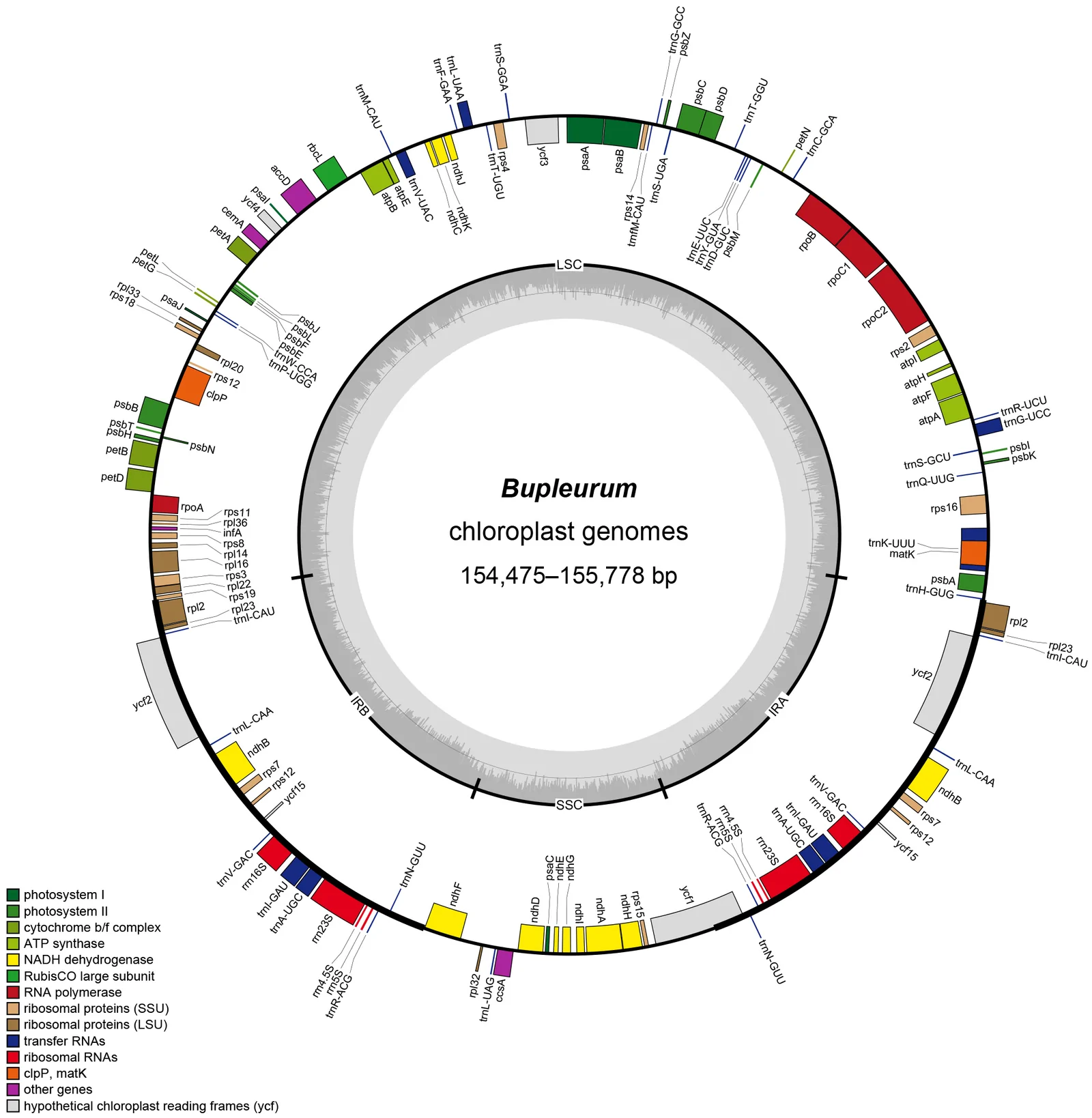
Circular map of the *Bupleurum* plastid genomes (154,496–155,778 bp). Genes drawn inside the circle are transcribed clockwise and those outside counter-clockwise; colours denote functional categories. The inner grey ring shows GC content, and the LSC, SSC and the two inverted repeats (IRa, IRb) are indicated.

All six assembled plastomes had the canonical quadripartite organisation of seed-plant plastid genomes (**Figure 3**; **Table 1**). Total length ranged from 154,496 bp in *B. diversifolium* to 155,778 bp in *B. falcatum* subsp. *cernuum*. The large single-copy region spanned 84,623–85,761 bp and the small single-copy region 17,277–17,573 bp, separated by two inverted repeats of 26,219–26,322 bp. Base composition was uniformly AT-rich, with overall GC content of 37.67–37.73%; the inverted repeats were slightly GC-enriched because they contain the ribosomal RNA operon.

**Table 1:** Characteristics of the six newly assembled *Bupleurum* plastid genomes.

Species	Size (bp)	LSC (bp)	IR (bp)	SSC (bp)	GC (%)	Genes	CDS	tRNA	rRNA
*Bupleurum arcticum*	155,677	85,536	26,305	17,531	37.71	132	87	37	8
*Bupleurum diversifolium*	154,496	84,623	26,298	17,277	37.73	132	87	37	8
*Bupleurum falcatum* subsp. *cernuum*	155,778	85,640	26,300	17,538	37.68	131	86	37	8
*Bupleurum longifolium*	155,475	85,330	26,315	17,515	37.70	132	87	37	8
*Bupleurum ranunculoides*	155,745	85,575	26,322	17,526	37.69	132	87	37	8
*Bupleurum ranunculoides* subsp. *telonense*	155,772	85,761	26,219	17,573	37.67	132	87	37	8

Each plastome encoded 131–132 annotated genes, comprising 86–87 protein-coding genes, 37 tRNA genes and eight rRNA genes (**Supplementary Table 2**). Six protein-coding genes (*rpl2*, *rpl23*, *ndhB*, *rps7*, *rps12* and *ycf2*), seven tRNAs and four rRNAs were duplicated in all six plastomes. Five plastomes also contained two *ycf15* annotations; *B. falcatum* subsp. *cernuum* lacked *ycf15* but contained an additional inverted-repeat-associated *ycf1* annotation, yielding a net total of 131 rather than 132 genes. Fifteen genes contained one intron, while *clpP*, *ycf3* and *rps12* contained two; the first exon of *rps12* was trans-spliced. Gene order and orientation were otherwise identical among the six plastomes.

### Codon usage bias and selective pressure

Codon usage was highly congruent among the six plastomes (**Figures 1A, B**; **Supplementary Table 3**). Among synonymous codons, A- or U-ending codons generally had RSCU values above 1, whereas most G- or C-ending alternatives were under-represented; UUG for leucine was the main G-ending exception (RSCU 1.30–1.31). The most strongly favoured codon was UUA for leucine (RSCU 1.78–1.79), followed by AGA for arginine (1.75–1.79), GCU for alanine (1.73–1.74) and UCU for serine (1.70–1.74). Methionine (AUG) and tryptophan (UGG), each encoded by a single codon, had RSCU = 1 as expected. The overall pattern is consistent with the AT-rich nucleotide composition of the plastomes.

Finite pairwise Ka/Ks estimates were below 1 for most plastid protein-coding genes (**Figure 1C**; **Supplementary Table 4**). Three of the 294 finite estimates exceeded 1, each in a single comparison: *rps14* in *B. diversifolium* (1.62), and *rpl2* (1.14) and *rpoA* (1.03) in *B. ranunculoides* subsp. *telonense*. Before *atpF* was re-annotated at the canonical group II splice site, that gene had produced five ratios above 1, three of them 1.60–1.63; none survived correction of the exon 1/exon 2 boundary. Because these are pairwise estimates among closely related plastomes and several genes had undefined or very small Ks estimates, ratios above 1 are treated as hypothesis-generating observations rather than formal evidence of positive selection.

Site-specific codon models applied to *atpF* and to the three genes whose pairwise ratios exceeded 1 gave no evidence of positive selection. Gene-wide ω was 0.36 for *atpF*, 0.24 for *rps14*, 0.36 for *rpoA* and 0.59 for *rpl2*. FEL detected a single site with β > α at p ≤ 0.05, in *atpF*, and MEME detected the same site and no other; across the 100–360 codons tested per gene this is far fewer than the 5–18 sites expected at this threshold by chance alone. FEL instead recovered 1–9 sites per gene with β significantly below α, consistent with purifying selection (**Supplementary Table 4**).

### Microsatellites and dispersed repeats

MISA detected 55–61 pure SSRs per plastome (346 in total), with *B. diversifolium* containing the most and *B. ranunculoides* the fewest (**Figure 1D**; **Supplementary Tables 5, S6**). Mononucleotide repeats dominated every genome (36–44 loci) and were almost entirely A/T motifs, whereas longer motif classes were less frequent. Compound SSRs are listed separately in **Supplementary Table 5** and are not included in the totals in **Supplementary Table 6** or **Figure 1D**. The concentration of A/T-rich SSRs in single-copy, intergenic and intronic regions is typical of angiosperm plastomes and provides candidate loci for length-polymorphism markers (Provan et al., 2001; Ebert and Peakall, 2009).

REPuter returned 41–50 forward or palindromic repeat pairs per plastome when the full-length inverted-repeat match was included (**Figure 1E**; **Supplementary Table 7**). Excluding repeat matches longer than 10 kb left 40–49 shorter pairs per plastome, most of them 30–50 bp long. Repeat content was therefore modest and broadly conservative, suggesting the absence of large rearrangements among the six plastomes.

### Sequence divergence and candidate molecular markers

The six-plastome comparison and the broader 51-accession genome-wide sequence comparison confirmed strong but uneven sequence conservation (**Figure 2A**; **Supplementary Figure 1**). Divergence was concentrated in the single-copy regions, the inverted repeats were comparatively conserved, and non-coding spacers were generally more variable than adjacent genes. In the 51-accession comparison, *B. gibraltaricum*, *B. petraeum* and *B. stellatum* were among the most divergent plastomes relative to the *B. chinense* reference.

Applying the composite 0–100 scoring scheme, MarkerSeek ranked 265 projected features across the 51-accession data set: 127 intergenic spacers, 85 protein-coding genes, 45 tRNAs and eight rRNAs. The highest-scoring candidates were intergenic spacers: *atpF–atpH* (89.7), *petA–psbJ* (84.2), *rpl32–trnL-UAG* (83.9), *petN–psbM* (80.8), *atpH–atpI* (77.5), *ndhF–rpl32* (77.1), *trnS-GCU–trnG-UCC* (75.8), *rps16–trnQ-UUG* (75.2) and *trnH-GUG–psbA* (74.6). The *atpF–atpH* spacer had the highest feature-level nucleotide diversity (π = 0.054), and *ycf1* was the most informative coding region (**Figure 2B**; **Supplementary Table 8**). Several candidates overlap hypervariable loci reported independently in *Bupleurum* and broader plant-barcode surveys (Dong et al., 2012; Chao et al., 2023).

### In silico candidate-barcode performance

**Figure 4:**
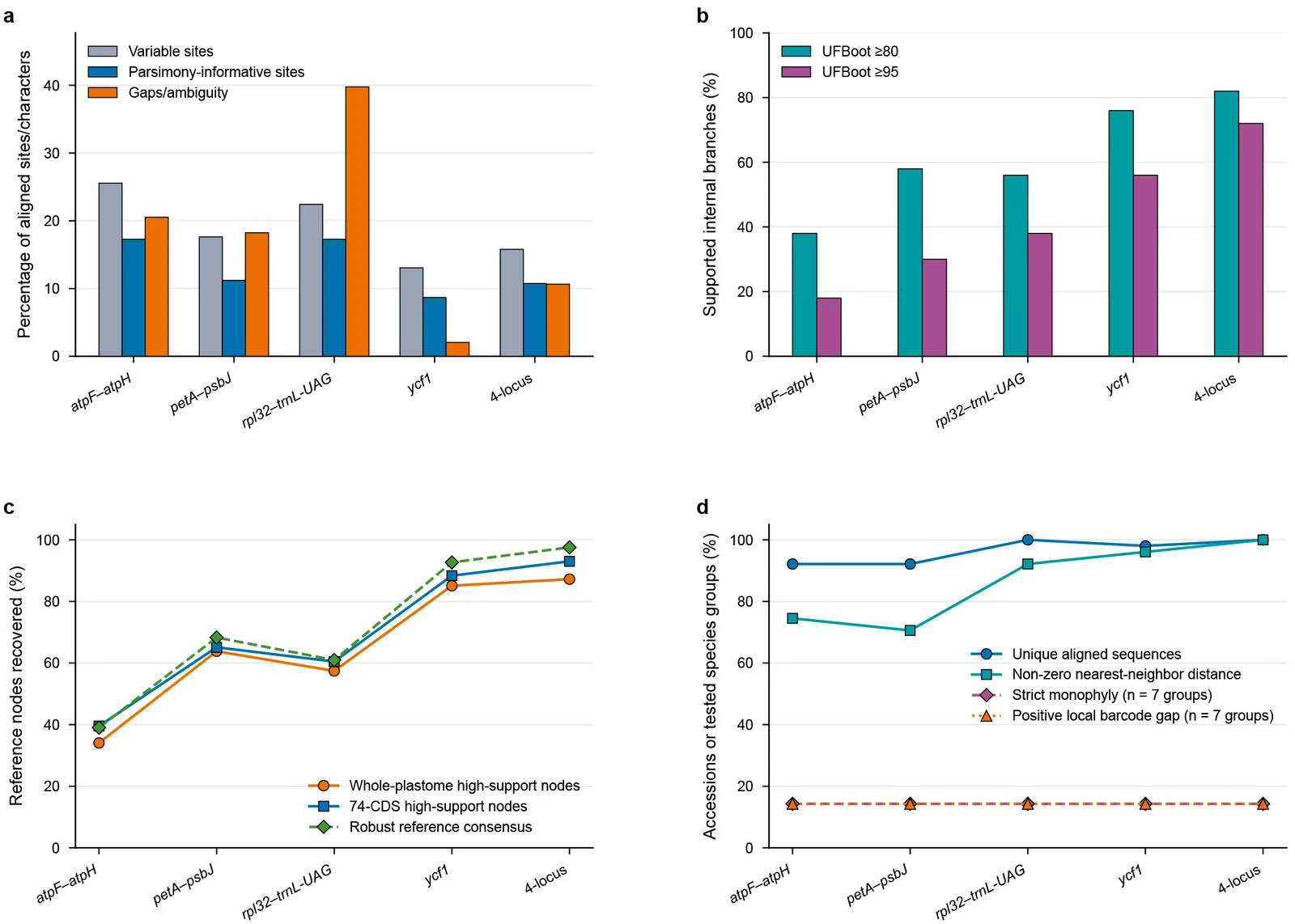
In silico phylogenetic resolution and candidate discriminatory performance of four plastid barcode regions in *Bupleurum*. **(A)** Ingroup variable-site and parsimony-informative-site percentages and the proportion of gaps or ambiguous characters. **(B)** Proportions of the 50 non-trivial branches in each 53-taxon maximum-likelihood tree supported at UFBoot ≥80 and ≥95. **(C)** Recovery of high-support nodes in the whole-plastome tree, the 74-CDS tree, and the robust reference consensus (nodes with UFBoot ≥95 in both reference trees). **(D)** Exact aligned-sequence uniqueness, non-zero nearest-neighbour distance, strict monophyly, and positive local barcode-gap performance. Discriminatory metrics use 51 *Bupleurum* accessions; monophyly and barcode gaps are restricted to seven operational binomial groups with more than one accession. The four-locus matrix was analysed under an edge-linked proportional four-partition model. These results represent in silico candidate performance, not validated species identification.

The four single-locus alignments comprised 544, 1,231, 1,146 and 5,596 bp for *atpF*–*atpH*, *petA*–*psbJ*, *rpl32*–*trnL-UAG* and *ycf1*, respectively, and contained 94, 138, 198 and 486 parsimony-informative ingroup sites. The corresponding proportions of gaps or ambiguous characters were 20.5%, 18.2%, 39.8% and 2.0%. Among individual loci, *ycf1* showed the strongest overall performance: it recovered 38 of 41 robust reference nodes and 28 of 50 internal branches at UFBoot ≥95, while 50 of 51 ingroup sequences were unique and 49 had a non-zero nearest-neighbour distance (**Figure 4**; **Supplementary Tables 10–S12**).

The 8,517-bp partitioned four-locus matrix contained 916 parsimony-informative sites and recovered 41 of 50 internal branches at UFBoot ≥80 and 36 at UFBoot ≥95. It shared 41 of 50 unrooted splits with the whole-plastome tree and 43 of 50 with the 74-CDS tree, recovered 40 of 41 robust reference nodes, and recovered all five focal relationships supported by both reference analyses. All 51 ingroup sequences were unique and had non-zero nearest-neighbour distances. However, among the seven operational binomial groups with multiple accessions, only one was strictly monophyletic and only one showed a positive local barcode gap. These results quantify in silico phylogenetic resolution and candidate discriminatory performance; they do not constitute validated species identification (**Figure 4**; **Supplementary Tables 10, S11**).

### Phylogenomic relationships

**Figure 5:**
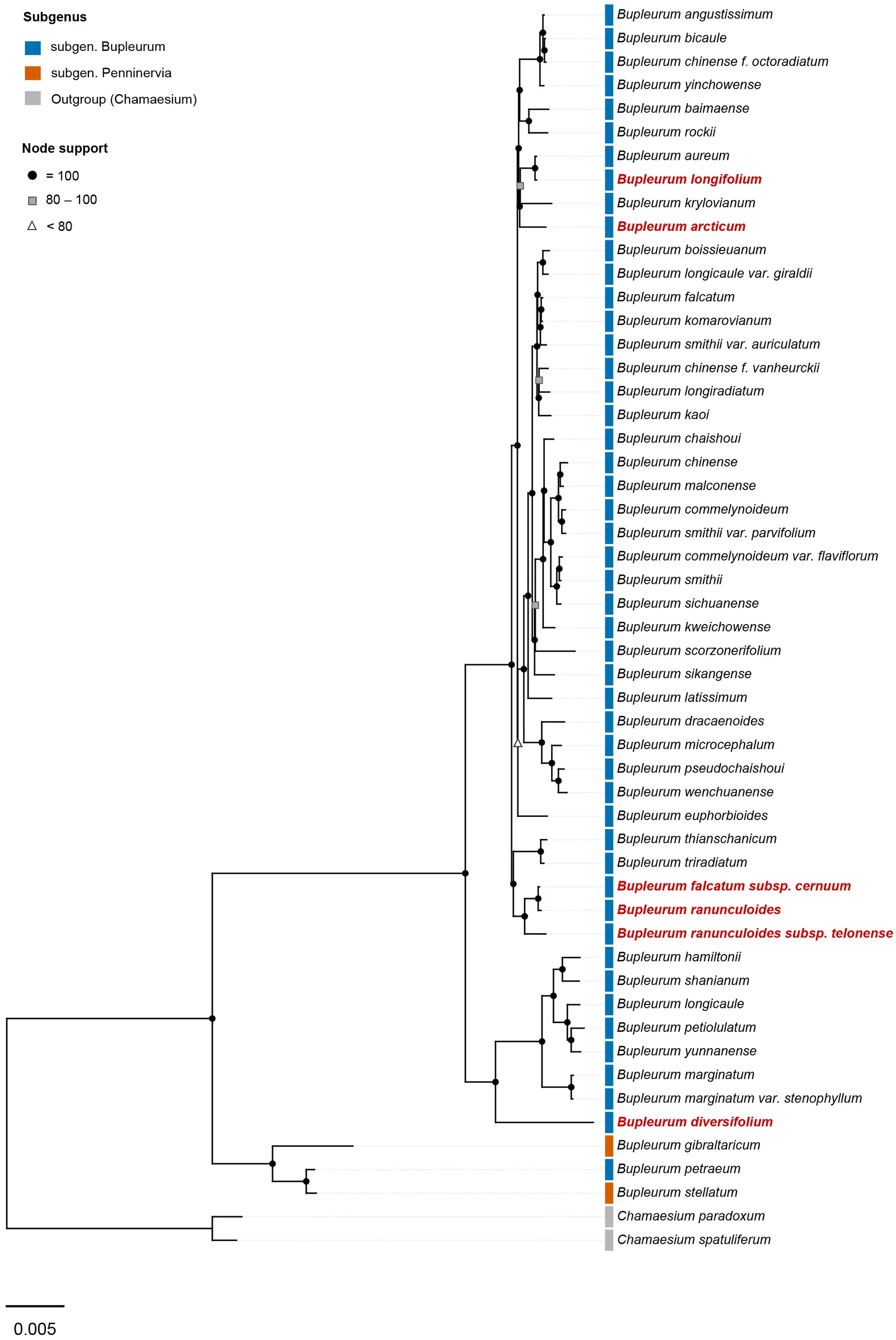
Maximum-likelihood phylogeny of *Bupleurum* inferred from 53 complete plastid genomes (193,173 aligned bp) under the TVM+F+R5 model. Node symbols summarise ultrafast-bootstrap support (circle = 100; square = 80–<100; triangle <80). Newly assembled taxa are shown in red, and the colour strip indicates the literature-based subgeneric assignments listed in **Supplementary Table 9** rather than inferred clades. *Chamaesium paradoxum* and *C. spatuliferum* were used as outgroups.

Maximum-likelihood analysis of 53 complete plastomes (51 *Bupleurum* and two *Chamaesium* outgroups) recovered *Bupleurum* as monophyletic relative to *Chamaesium* (UFBoot = 100%; **Figure 5**). The first ingroup split separated *B. gibraltaricum* and the sister pair *B. petraeum–B. stellatum from the remaining accessions* (UFBoot = 100%). Because these taxa span the two literature-based assignments used in **Supplementary Table 9**, the two sampled accessions assigned to *Penninervia* did not form an exclusive clade in the present tree. With only two *Penninervia* accessions included, this topology is reported as a sampling-limited phylogenetic observation rather than as a rejection of traditional subgeneric classification. An eight-taxon lineage containing *B. diversifolium* then branched before the remaining core radiation.

The six newly assembled taxa were placed in several distinct parts of the plastid tree. *B. falcatum* subsp. *cernuum* was sister to *B. ranunculoides*, and *B. ranunculoides* subsp. *telonense* was sister to that pair rather than to *B. ranunculoides* alone. *B. longifolium* was sister to *B. aureum* within a four-taxon lineage that also contained *B. krylovianum* and *B. arcticum*. These placements show that the new accessions do not constitute a single lineage and that the plastid data alone do not corroborate the expected immediate sister relationship between *B. ranunculoides* and subsp. *telonense*. The partitioned 74-CDS tree recovered all of these key placements with UFBoot = 100% (**Supplementary Figure 2**). Overall, the whole-plastome and CDS trees shared 45 of 50 internal bipartitions (90%); the five differences were restricted to short internal branches within the core East Asian radiation.

### Candidate plastid RNA-editing predictions in the six newly assembled plastomes

The PREP-Cp-compatible screen analysed all 35 supported genes in each of the six plastomes and predicted 367 nonsynonymous C-to-U candidates (**Supplementary Table 13**). Counts ranged from 57 in *Bupleurum diversifolium* to 63 in *B. falcatum* subsp. *cernuum*, *B. ranunculoides* and *B. ranunculoides* subsp. *telonense*; *B. arcticum* and *B. longifolium* had 61 and 60, respectively. Predictions occurred in 21 genes in *B. diversifolium* and 22 genes in each of the other accessions.

Across accessions, 269 candidates (73.3%) affected the second codon position and 98 (26.7%) the first; none affected the third because the workflow reports only amino-acid-changing events. The greatest numbers occurred in *ndhB* (60 candidates), *matK* (36) and *rpoB* (35). The most frequent predicted amino-acid changes were serine to leucine (99), proline to leucine (59) and serine to phenylalanine (41). Scores were 1.00 for 266 candidates, 0.86 for 74 and 0.80 for 27.

## Discussion

The six plastomes reported here reinforce a picture of strong architectural stability in *Bupleurum*. Genome size, quadripartite structure, GC content and gene inventory varied only slightly, gene order was invariant, and the annotation differences involved the short *ycf15* reading frame and an inverted-repeat-associated *ycf1* copy rather than rearrangement or loss of an essential gene. This conservatism is shared with published *Bupleurum* and Apiaceae plastomes (Huang et al., 2021a; Weng et al., 2023) and explains how the same data can support both deep phylogenetic inference and fine-scale marker discovery: a slowly evolving, collinear backbone coexists with a limited number of variable single-copy spacers.

The Ka/Ks analysis adds a functional dimension that has rarely been examined in the genus (Huang et al., 2021a). The genome-wide signal of purifying selection is expected for plastid genes that encode core components of photosynthesis and gene expression, whereas the ratios above 1 require caution (Mugal et al., 2013). The clearest case proved instructive: *atpF* originally produced five ratios above 1, three of them 1.60–1.63, but that signal was an annotation artefact. In all six new plastomes the *atpF* exon 1/exon 2 junction had been placed 18 bp downstream of the canonical group II 3′ splice site, so six codons of intron were compared as if they were coding sequence; after re-annotation no *atpF* ratio exceeded 1, and the gene-wide ω estimated across all 53 taxa fell from 0.70 to 0.36. The three ratios that remain above 1 each rest on a single comparison. Such ratios can reflect statistical inflation of Ka/Ks that commonly arises among very closely related taxa when synonymous substitutions are too few to estimate Ks reliably (Mugal et al., 2013). Accordingly, the present pairwise estimates do not demonstrate positive selection. Site-specific codon models — FEL and MEME (Kosakovsky Pond and Frost, 2005; Murrell et al., 2012) — were therefore applied to *atpF* and to the three genes whose ratios remained above 1. Neither test recovered more sites under positive selection than the number expected by chance at the same threshold, whereas FEL recovered sites under significant purifying selection in every gene (**Supplementary Table 4**). We therefore find no evidence of positive selection on plastid protein-coding genes in the genus, and note that comparative Ka/Ks screens of plastomes are sensitive to inconsistencies in intron annotation.

Translating plastome variation into practical markers is the step at which many comparative studies stop short, typically reporting a sliding-window diversity plot without an explicit ranking criterion (Huang et al., 2021a; Weng et al., 2023). By scoring every annotated feature on diversity, alignment reliability, flanking conservation, species resolution and length, MarkerSeek converts the divergence landscape into a reproducible, ranked candidate list (Gao, 2025). The loci it placed at the top of that list—*atpF*–*atpH*, *petA*–*psbJ*, *rpl32*–*trnL*-UAG, *petN*–*psbM*, *atpH*–*atpI* and *ycf1*—are biologically sensible: all lie in the single-copy regions, most are intergenic, and several recur as hypervariable hotspots in independent *Bupleurum* and broader angiosperm barcoding studies (Dong et al., 2012; Gou et al., 2020). This concordance is reassuring, but the value of the present screen is that it also supplies, for each region, the quantitative evidence and conserved priming flanks needed to move directly to primer design. Subsequent phylogenetic tests nevertheless showed clear performance differences: *ycf1* was the strongest single locus, whereas the partitioned four-locus matrix raised the proportion of branches with UFBoot ≥95 to 72% and recovered 40 of 41 robust reference nodes.

These candidates contain more variation in the present alignment than the universal plastid barcodes *rbcL* and *matK* (CBOL Plant Working Group, 2009). Conventional plastid loci can be too conserved to resolve congeneric *Bupleurum* species (Chao et al., 2023). They are also relevant to applied identification because Radix Bupleuri is frequently adulterated or substituted, and reliable molecular authentication underpins safety and quality control (Teng et al., 2023). The partitioned four-locus matrix distinguished all 51 accession sequences, but this accession-level result is not a species-identification test. A short panel of high-ranking plastid spacers, or the complete plastome used as a super-barcode (Wu et al., 2021; Liu et al., 2024), may therefore support authentication and germplasm management. However, the present validation remains in silico: only seven operational binomial groups had multiple accessions, only one was strictly monophyletic, and only one showed a positive local barcode gap. These candidates therefore warrant population-level sampling, primer testing and blinded identification trials before diagnostic performance can be claimed (Meyer and Paulay, 2005).

The whole-plastome phylogeny and the partitioned CDS sensitivity analysis both support the monophyly of *Bupleurum* relative to the two *Chamaesium* outgroups (Huang et al., 2021a). Under the literature-based assignments used in **Supplementary Table 9**, the two sampled accessions of subgenus *Penninervia* (*B. gibraltaricum* and *B. stellatum*) occupy early-diverging positions but do not form an exclusive clade by themselves, and *B. petraeum* (assigned to subgenus *Bupleurum*) clusters with them (Neves and Watson, 2004). We report this pattern as a phylogenetic observation within the present sampling rather than as evidence that the traditional two-subgenus classification is invalid. Only two accessions currently assigned to *Penninervia* are included, literature assignments may themselves be incomplete or uneven across publicly available plastomes, and a single maternal genealogy can be distorted by plastid introgression or incomplete lineage sorting (Song et al., 2024). Formal evaluation or revision of infrageneric classification would require denser sampling of both subgenera, morphological re-evaluation and independent nuclear loci (Neves and Watson, 2004). The two plastid analyses shared 90% of their internal bipartitions and all focal relationships involving the six new accessions, whereas the limited differences occurred among short-branched lineages in the core radiation. The CDS analysis is less sensitive to inconsistent circular genome start positions, SSC orientation and non-coding alignment, while the whole-plastome analysis includes additional intergenic signal; their agreement therefore supports the stable parts of the plastid genealogy. Within the newly sampled taxa, the sister relationship between *B. falcatum* subsp. *cernuum* and *B. ranunculoides*, followed by subsp. *telonense*, means that this plastid genealogy does not by itself support the anticipated *ranunculoides–telonense* pairing.

Two cautions temper these conclusions. First, because plastids are maternally inherited, the tree records a single, uniparental genealogy; in a young and rapidly diversifying group such as East Asian *Bupleurum*, hybridisation and incomplete lineage sorting can cause the plastome history to depart from the species tree, and the short internal branches that underlie our few weakly supported nodes are precisely where such conflict is expected (Song et al., 2024). Second, even 51 *Bupleurum* accessions sample only a fraction of the genus, and the East Asian radiation remains incompletely represented, so the backbone presented here should be regarded as a robust but still incomplete scaffold to be tested with nuclear data and broader taxon sampling (Song et al., 2024).

The comparative screen yielded closely similar profiles across the six plastomes, with 57–63 predicted sites in 21–22 genes per accession. This per-accession range is comparable to the 56 PREP-Cp candidates reported for each of seven *Ligusticum* plastomes (Yuan et al., 2021), but direct numerical comparisons remain method-dependent because prediction-based and transcript-derived surveys differ in gene coverage and in whether synonymous or low-efficiency events are counted (Small et al., 2020). Within our data, 269 of 367 candidates (73.3%) affected the second codon position, and the dominant predicted changes (serine to leucine, proline to leucine, and serine to phenylalanine) would replace serine or proline with hydrophobic residues, consistent with the protein-restorative bias described across vascular-plant plastid editomes (Duan et al., 2023). The concentration of candidates in *ndhB*, *matK* and *rpoB* further shows that the predicted sites are unevenly distributed among plastid genes. At the DNA level, plant organellar evolution is thought to reflect interacting effects of mutation, repair, selection and drift rather than a single process (Wang et al., 2024). This broader framework reinforces the need to separate plastome conservation from conservation of RNA processing: similar genomic sequences do not establish identical editing states, which additionally depend on nuclear-encoded specificity factors and biological context (Lenz et al., 2018). Accordingly, the similarity among accessions reflects both biological conservation and the conservation-based scoring rule; it does not demonstrate an experimentally conserved *Bupleurum* editome (Mower, 2009; Small et al., 2020).

The 367 entries are computational hypotheses rather than observed editing events (Mower, 2009). Because the PREP-Cp-compatible procedure scores only nonsynonymous C-to-U changes in 35 genes, it cannot recover synonymous events, sites outside this gene set or editing efficiencies (Mower, 2009). Exact web-server replication was impossible because the original manually adjusted alignments are unavailable, and the local regression check recovered one of the two sites in the archived *Vitis accD* example. The site list is therefore most appropriate for prioritising RNA-seq or targeted cDNA sequencing, not for defining an editome. Claims of cross-accession conservation or functional consequence will require site-level validation in all six accessions using comparable tissues, developmental stages and environmental conditions, followed where relevant by quantitative estimates of editing efficiency (Small et al., 2020).

## Conclusions

We present six newly assembled *Bupleurum* plastomes and integrate them with a data set of 45 published *Bupleurum* plastomes. The plastomes are structurally conservative but contain localised divergence suitable for candidate-marker development; MarkerSeek ranks a primer-oriented panel led by *atpF–atpH*, *petA–psbJ*, *rpl32–trnL-UAG* and *ycf1*. In silico tests identified *ycf1* as the strongest single locus and the partitioned four-locus matrix as the analysis most congruent with the reference trees, while sparse intraspecific sampling precludes a validated species-identification claim. Concordant 53-taxon whole-plastome and 74-CDS genealogies support genus monophyly. Within the present sampling they do not recover the two literature-based subgeneric assignments as exclusive reciprocal lineages, but the sparse representation of subgenus *Penninervia* and the maternal inheritance of plastids preclude a taxonomic rejection of the traditional classification. The analyses also place *B. falcatum* subsp. *cernuum* with *B. ranunculoides* rather than recovering the anticipated immediate ranunculoides–telonense pairing. Finally, the PREP-Cp-compatible analysis predicted 367 nonsynonymous C-to-U candidates across the six new plastomes, providing a reproducible hypothesis set for transcript-level validation rather than an experimentally established editome. These results provide genomic resources while defining phylogenetic and RNA-editing hypotheses that require nuclear and experimental validation.

## Data Availability

GenBank accession numbers PZ518415–PZ518420 have been assigned to the six plastid genomes. Assembly-read SRA accessions are listed in **Supplementary Table 1**. Site-level RNA-editing predictions, accessions and analysis parameters are provided in **Supplementary Table 13**; scripts, reference manifests and per-gene alignments are available from the corresponding author on reasonable request. Other supporting data are included in the article and its additional files.

## Supplementary material

Supplementary Figure 1 Genome-wide sequence identity across 51 *Bupleurum* plastomes, using *B. chinense* as the reference. The horizontal axis is plastome position and the vertical axis is sequence identity (50–100%).

Supplementary Figure 2 Maximum-likelihood plastid CDS phylogeny of *Bupleurum* inferred from 74 protein-coding genes (66,250 aligned bp) in 53 accessions using a partitioned model. Numbers at nodes are ultrafast-bootstrap percentages; values below 50 are omitted. Newly assembled accessions are shown in red, and the colour strip indicates the literature-based subgeneric assignments listed in **Supplementary Table 9**. The terminal order was rotated to match **Figure 5** without changing topology; *Chamaesium paradoxum* and *C. spatuliferum* are displayed at the bottom as outgroups.

Supplementary Figure 3 Maximum-likelihood phylogeny inferred from *atpF–atpH* for 51 *Bupleurum* accessions and two *Chamaesium* outgroups. The 544-bp alignment was analysed under TVM+F+G4 with 1,000 ultrafast-bootstrap replicates. Node symbols indicate UFBoot support (blue, ≥95; orange, 80–94; open grey, <80). Coloured squares denote the taxonomic groups shown in the legend, and the six newly sequenced plastomes are highlighted in red. Branch lengths are substitutions per site.

Supplementary Figure 4 Maximum-likelihood phylogeny inferred from *petA–psbJ* for 51 *Bupleurum* accessions and two *Chamaesium* outgroups. The 1,231-bp alignment was analysed under K3Pu+F+G4 with 1,000 ultrafast-bootstrap replicates. Node symbols indicate UFBoot support (blue, ≥95; orange, 80–94; open grey, <80). Coloured squares denote the taxonomic groups shown in the legend, and the six newly sequenced plastomes are highlighted in red. Branch lengths are substitutions per site.

Supplementary Figure 5 Maximum-likelihood phylogeny inferred from *rpl32–trnL-UAG* for 51 *Bupleurum* accessions and two *Chamaesium* outgroups. The 1,146-bp alignment was analysed under K3Pu+F+R3 with 1,000 ultrafast-bootstrap replicates. Node symbols indicate UFBoot support (blue, ≥95; orange, 80–94; open grey, <80). Coloured squares denote the taxonomic groups shown in the legend, and the six newly sequenced plastomes are highlighted in red. Branch lengths are substitutions per site.

Supplementary Figure 6 Maximum-likelihood phylogeny inferred from *ycf1* for 51 *Bupleurum* accessions and two *Chamaesium* outgroups. The 5,596-bp alignment was analysed under K3Pu+F+R2 with 1,000 ultrafast-bootstrap replicates. Node symbols indicate UFBoot support (blue, ≥95; orange, 80–94; open grey, <80). Coloured squares denote the taxonomic groups shown in the legend, and the six newly sequenced plastomes are highlighted in red. Branch lengths are substitutions per site.

Supplementary Figure 7 Maximum-likelihood phylogeny inferred from the partitioned four-locus matrix for 51 *Bupleurum* accessions and two *Chamaesium* outgroups. The 8,517-bp matrix was analysed under an edge-linked proportional model with TPM3+F+R2 for *atpF–atpH*, K3Pu+F+G4 for *petA–psbJ*, K3Pu+F+R2 for *rpl32–trnL-UAG* and K3Pu+F+R2 for *ycf1*, with 1,000 ultrafast-bootstrap replicates. Node symbols indicate UFBoot support (blue, ≥95; orange, 80–94; open grey, <80). Coloured squares denote the taxonomic groups shown in the legend, and the six newly sequenced plastomes are highlighted in red. Branch lengths are substitutions per site.
