# Supplementary material for: Comparative chloroplast genomics of six Bupleurum (Apiaceae) accessions: candidate barcodes, phylogeny based on available plastomes, and candidate RNA-editing sites
Source: Front Plant Sci. 2026 Aug 20;17:1916637. doi: 10.3389/fpls.2026.1916637 (PMC13538832; doi:10.3389/fpls.2026.1916637)
Supplement: Supplementary file 1 [file DataSheet1.pdf]

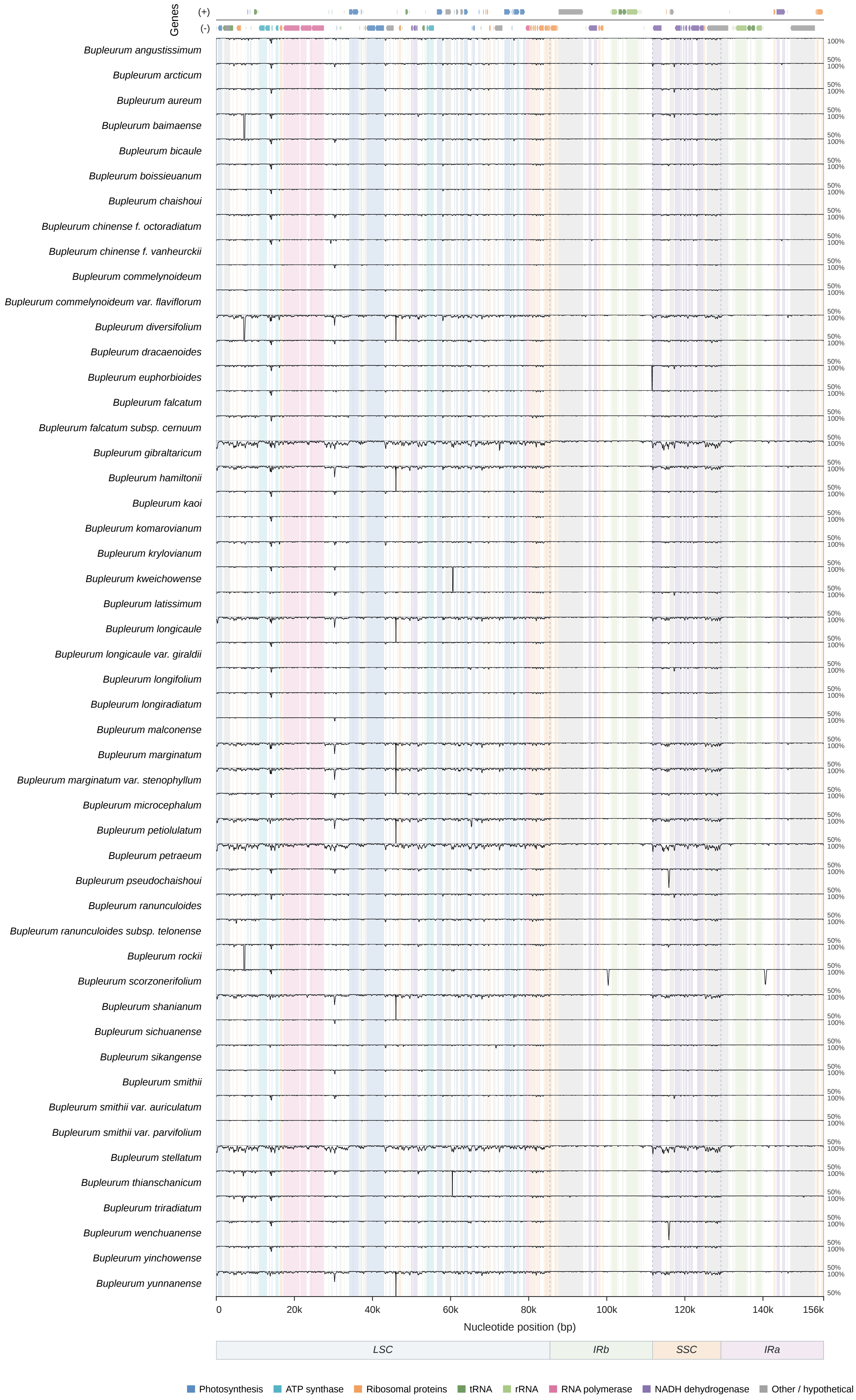
Genes
(+)
(-)
Bupleurum angustissimum
Bupleurum arcticum
Bupleurum aureum
Bupleurum baimaense
Bupleurum bicaule
Bupleurum boissieuanum
Bupleurum chaishoui
Bupleurum chinense f. octoradiatum
Bupleurum chinense f. vanheurckii
Bupleurum commelynoideum
Bupleurum commelynoideum var. flaviflorum
Bupleurum diversifolium
Bupleurum dracaenoides
Bupleurum euphorbioides
Bupleurum falcatum
Bupleurum falcatum subsp. cernuum
Bupleurum gibraltaricum
Bupleurum hamiltonii
Bupleurum kaoi
Bupleurum komarovianum
Bupleurum krylovianum
Bupleurum kweichowense
Bupleurum latissimum
Bupleurum longicaule
Bupleurum longicaule var. giraldii
Bupleurum longifolium
Bupleurum longiradiatum
Bupleurum malconense
Bupleurum marginatum
Bupleurum marginatum var. stenophyllum
Bupleurum microcephalum
Bupleurum petiolulatum
Bupleurum petraeum
Bupleurum pseudochaishoui
Bupleurum ranunculoides
Bupleurum ranunculoides subsp. telonense
Bupleurum rockii
Bupleurum scorzonerifolium
Bupleurum shanianum
Bupleurum sichuanense
Bupleurum sikangense
Bupleurum smithii
Bupleurum smithii var. auriculatum
Bupleurum smithii var. parvifolium
Bupleurum stellatum
Bupleurum thianschanicum
Bupleurum triradiatum
Bupleurum wenchuanense
Bupleurum yinchowense
Bupleurum yunnanense
100%
50%
0
20k
40k
60k
80k
100k
120k
140k
156k
Nucleotide position (bp)
LSC
IRb
SSC
IRa
Photosynthesis
ATP synthase
Ribosomal proteins
tRNA
rRNA
RNA polymerase
NADH dehydrogenase
Other / hypothetical
