# Supplementary material for: Comparative chloroplast genomics of six Bupleurum (Apiaceae) accessions: candidate barcodes, phylogeny based on available plastomes, and candidate RNA-editing sites
Source: Front Plant Sci. 2026 Aug 20;17:1916637. doi: 10.3389/fpls.2026.1916637 (PMC13538832; doi:10.3389/fpls.2026.1916637)
Supplement: Supplementary file 2 [file DataSheet2.pdf]

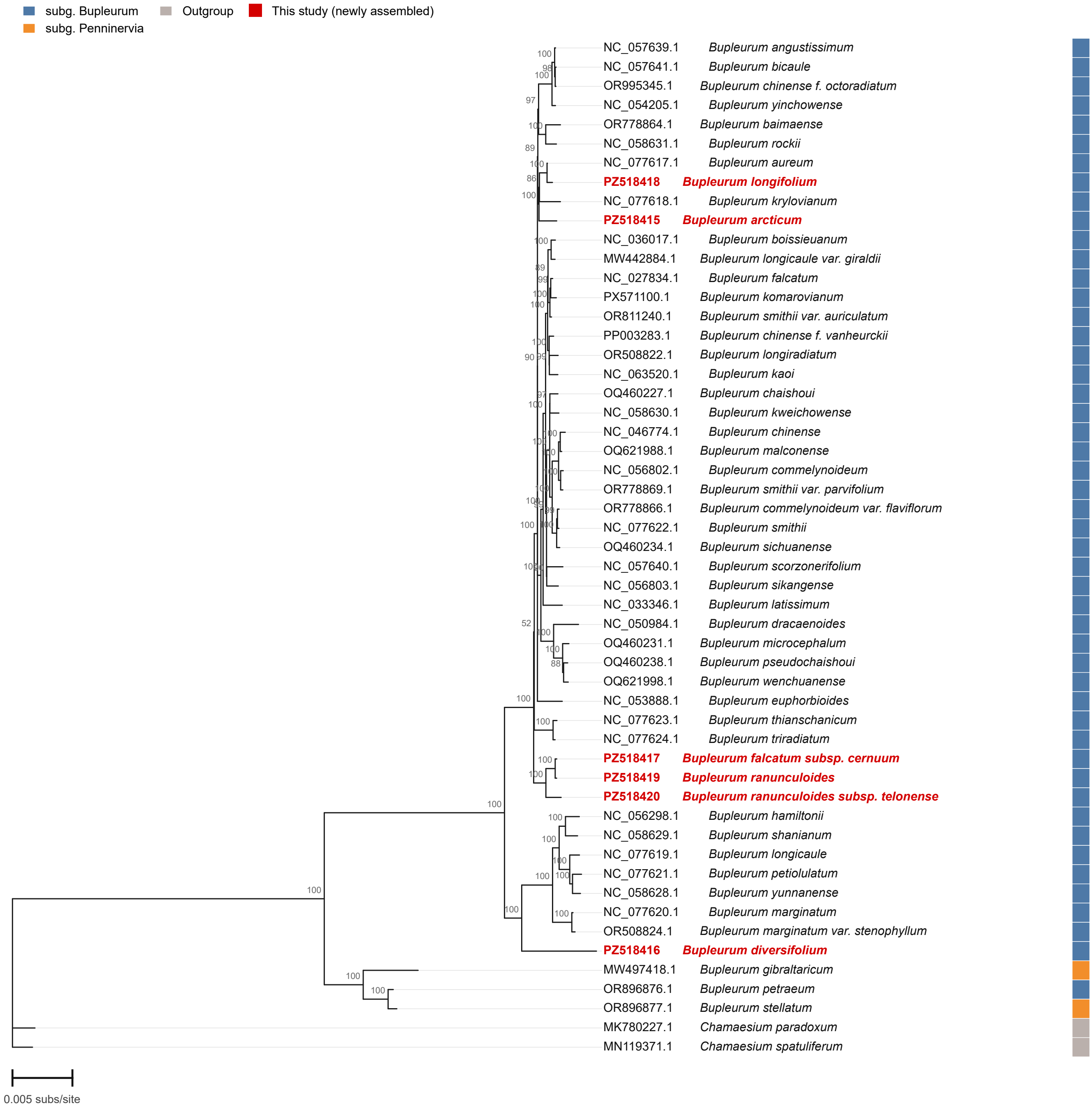

Maximum-likelihood phylogeny of *Bupleurum* (plastid CDS supermatrix)
