# Supplementary material for: Comparative chloroplast genomics of six Bupleurum (Apiaceae) accessions: candidate barcodes, phylogeny based on available plastomes, and candidate RNA-editing sites
Source: Front Plant Sci. 2026 Aug 20;17:1916637. doi: 10.3389/fpls.2026.1916637 (PMC13538832; doi:10.3389/fpls.2026.1916637)
Supplement: Supplementary file 5 [file DataSheet5.pdf]

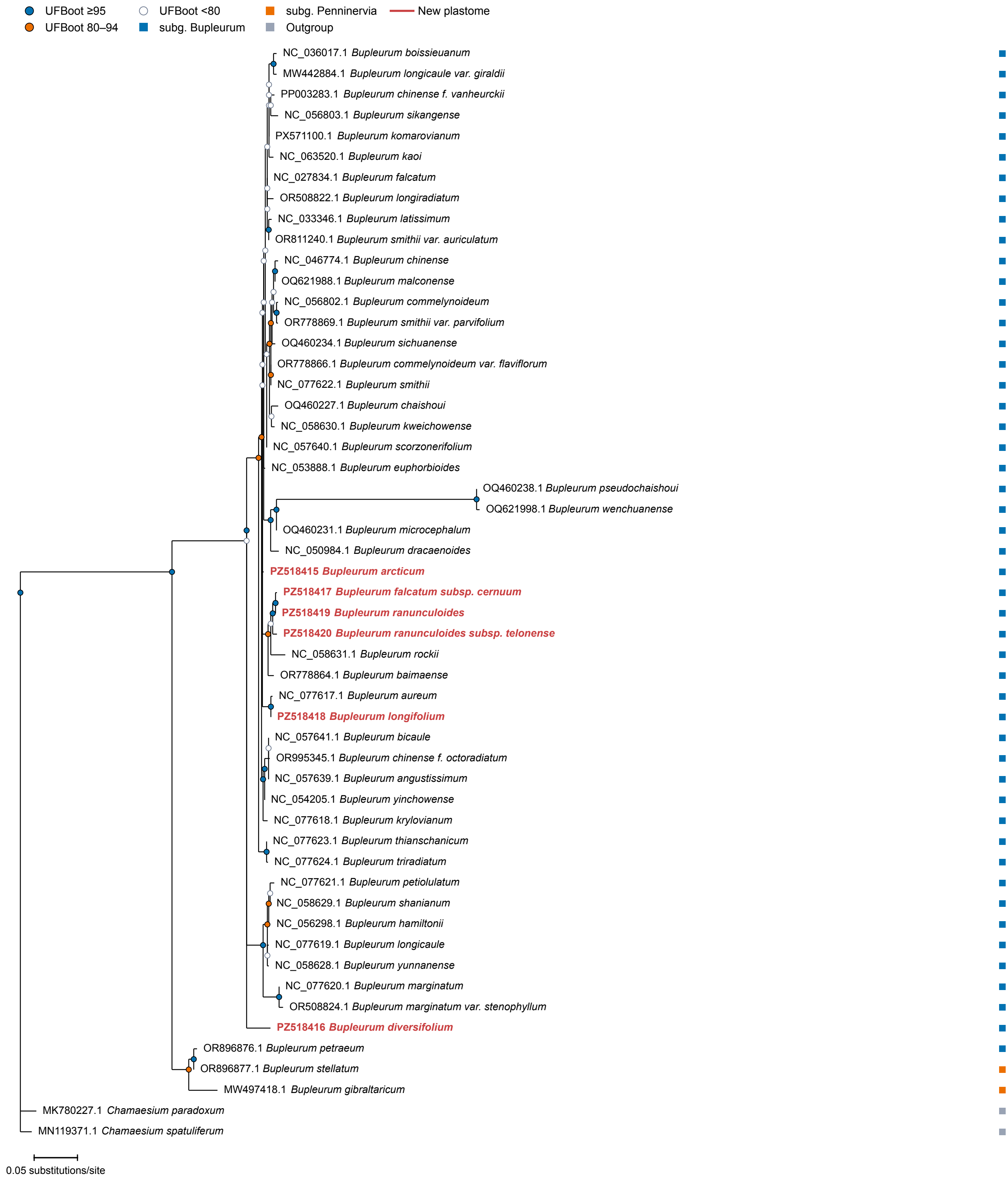
UFBoot ≥95
UFBoot 80–94
UFBoot <80
subg. Bupleurum
subg. Penninervia
Outgroup
New plastome
NC_036017.1 Bupleurum boissieuanum
MW442884.1 Bupleurum longicaule var. giraldii
PP003283.1 Bupleurum chinense f. vanheurckii
NC_056803.1 Bupleurum sikangense
PX571100.1 Bupleurum komarovianum
NC_063520.1 Bupleurum kaoi
NC_027834.1 Bupleurum falcatum
OR508822.1 Bupleurum longiradiatum
NC_033346.1 Bupleurum latissimum
OR811240.1 Bupleurum smithii var. auriculatum
NC_046774.1 Bupleurum chinense
OQ621988.1 Bupleurum malconense
NC_056802.1 Bupleurum commelynoideum
OR778869.1 Bupleurum smithii var. parvifolium
OQ460234.1 Bupleurum sichuanense
OR778866.1 Bupleurum commelynoideum var. flaviflorum
NC_077622.1 Bupleurum smithii
OQ460227.1 Bupleurum chaishoui
NC_058630.1 Bupleurum kweichowense
NC_057640.1 Bupleurum scorzonerifolium
NC_053888.1 Bupleurum euphorbioides
OQ460238.1 Bupleurum pseudochaishoui
OQ621998.1 Bupleurum wenchuanense
OQ460231.1 Bupleurum microcephalum
NC_050984.1 Bupleurum dracaenoides
PZ518415 Bupleurum arcticum
PZ518417 Bupleurum falcatum subsp. cernuum
PZ518419 Bupleurum ranunculoides
PZ518420 Bupleurum ranunculoides subsp. telonense
NC_058631.1 Bupleurum rockii
OR778864.1 Bupleurum baimaense
NC_077617.1 Bupleurum aureum
PZ518418 Bupleurum longifolium
NC_057641.1 Bupleurum bicaule
OR995345.1 Bupleurum chinense f. octoradiatum
NC_057639.1 Bupleurum angustissimum
NC_054205.1 Bupleurum yinchowense
NC_077618.1 Bupleurum krylovianum
NC_077623.1 Bupleurum thianschanicum
NC_077624.1 Bupleurum triradiatum
NC_077621.1 Bupleurum petiolulatum
NC_058629.1 Bupleurum shanianum
NC_056298.1 Bupleurum hamiltonii
NC_077619.1 Bupleurum longicaule
NC_058628.1 Bupleurum yunnanense
NC_077620.1 Bupleurum marginatum
OR508824.1 Bupleurum marginatum var. stenophyllum
PZ518416 Bupleurum diversifolium
OR896876.1 Bupleurum petraeum
OR896877.1 Bupleurum stellatum
MW497418.1 Bupleurum gibraltaricum
MK780227.1 Chamaesium paradoxum
MN119371.1 Chamaesium spatuliferum
0.05 substitutions/site
